# Clinical and functional impact of TARBP2 over-expression in adrenocortical carcinoma

**DOI:** 10.1530/ERC-13-0098

**Published:** 2013-08

**Authors:** Stefano Caramuta, Linkiat Lee, Deniz M Özata, Pinar Akçakaya, Hong Xie, Anders Höög, Jan Zedenius, Martin Bäckdahl, Catharina Larsson, Weng-Onn Lui

**Affiliations:** 1 Department of Oncology-PathologyKarolinska InstitutetStockholmSweden; 2 Department of Molecular Medicine and SurgeryKarolinska InstitutetStockholmSweden; 3 Cancer Center Karolinska (CCK), R8:04Karolinska University Hospital-SolnaSE-17176, StockholmSweden; 4 Department of Breast and Endocrine SurgeryKarolinska University HospitalStockholmSweden

**Keywords:** TARBP2, over-expression, adrenocortical cancer, diagnostic, function

## Abstract

Deregulation of microRNA (miRNA) expression in adrenocortical carcinomas (ACCs) has been documented to have diagnostic, prognostic, as well as functional implications. Here, we evaluated the mRNA expression of *DROSHA*, *DGCR8*, *DICER* (*DICER1*), *TARBP2*, and *PRKRA*, the core components in the miRNA biogenesis pathway, in a cohort of 73 adrenocortical tumors (including 43 adenomas and 30 carcinomas) and nine normal adrenal cortices using a RT-qPCR approach. Our results show a significant over-expression of *TARBP2*, *DICER*, and *DROSHA* in the carcinomas compared with adenomas or adrenal cortices (*P*<0.001 for all comparisons). Using western blot and immunohistochemistry analyses, we confirmed the higher expression of TARBP2, DICER, and DROSHA at the protein level in carcinoma cases. Furthermore, we demonstrate that mRNA expression of *TARBP2*, but not *DICER* or *DROSHA*, is a strong molecular predictor to discriminate between adenomas and carcinomas. Functionally, we showed that inhibition of TARBP2 expression in human NCI-H295R ACC cells resulted in a decreased cell proliferation and induction of apoptosis. TARBP2 over-expression was not related to gene mutations; however, copy number gain of the *TARBP2* gene was observed in 57% of the carcinomas analyzed. In addition, we identified that *miR-195* and *miR-497* could directly regulate TARBP2 and DICER expression in ACC cells. This is the first study to demonstrate the deregulation of miRNA-processing factors in adrenocortical tumors and to show the clinical and biological impact of TARBP2 over-expression in this tumor type.

## Introduction

Adrenocortical tumors are detected in up to 7% of the population and are often found as incidental tumors (Hammarstedt *et al*. 2010). The majority of these tumors are adenomas while only a small proportion are classified as adrenocortical carcinomas (ACCs) with an annual incidence of two cases per million among adults worldwide (Schteingart 2007).

The prognosis for ACCs is poor with reported 5-year survival rates between 20 and 45%, and treatment alternatives are very limited (Allolio & Fassnacht 2006, Bertherat *et al*. 2006). In addition, discrimination of ACCs from adrenocortical adenomas can be challenging. Generally, a tumor is unambiguously classified as ACC when it presents signs of invasion and/or distant metastases. In the absence of metastasis, the diagnosis relies on the histopathological evaluation, and the histological Weiss score classification is still the most commonly used one (Weiss *et al*. 1989). Given the limitation of conventional histopathology in adrenocortical tumor diagnostics, the identification of additional molecular markers with diagnostic and prognostic potential for clinical management of ACC is needed.

Previous reports identified over-expression of IGF2, loss-of-heterozygosity at 11p15 and 17p13, and increased MIB-1 proliferation index as molecular biomarkers to distinguish adrenocortical adenomas and carcinomas (Yano *et al*. 1989, Ilvesmaki *et al*. 1993, Gicquel *et al*. 1994, Stojadinovic *et al*. 2003). Several studies have shown the potential of mRNA and microRNA (miRNA) profiling to correctly classify adenomas or carcinomas and to identify subgroups of ACC patients with different survival and outcome (de Fraipont *et al*. 2005, Giordano *et al*. 2009, Laurell *et al*. 2009, de Reynies *et al*. 2009, Soon *et al*. 2009, Tombol *et al*. 2009, Özata *et al*. 2011, Patterson *et al*. 2011, Schmitz *et al*. 2011).

While the role of miRNAs in cancer development and progression is established, recent studies are also focusing on the deregulation of miRNA-processing factors, which are needed for miRNA maturation. This pathway has been shown to play an important role in tumor initiation and progression and to have a prognostic potential in several cancer types (Roh *et al*. 2005, Sugito *et al*. 2006, Chiosea *et al*. 2007, Merritt *et al*. 2008, Melo *et al*. 2009).

In this study, we evaluated the expression of the miRNA machinery components as potential diagnostic and prognostic markers in adrenocortical tumors. We further investigated the effect of TARBP2 deregulation on cell growth and apoptosis and explored possible mechanisms responsible for its deregulation in this tumor type.

## Materials and methods

### Clinical material

This study included 73 snap–frozen primary sporadic adrenocortical tumors from 72 patients and nine histopathologically verified normal adrenal cortices. All tissue samples were stored at −80 °C until use. Representative sections from all specimens were subjected to histopathological evaluation to confirm more than 80% tumor cells in the samples. Tumors were histopathologically classified according to the WHO classification (DeLellis *et al*. 2004). The diagnosis of ACC was based on vascular invasion, nuclear grade, mitotic index, tumor necrosis, invasion of surrounding organs, and/or presence of distant metastasis. Clinical details of tumor cases have been partially published in previous studies (Laurell *et al*. 2009, Özata *et al*. 2011) and are summarized in Table 1. All samples were collected with informed consent, and the study of tissue material was approved by the local ethics committee (Dnr 01-353,01-136).

### Cell line

The ACC cell line NCI-H295R was purchased from the American Type Culture Collection (LGC Standards, Middlesex, UK) and maintained in culture as described previously (Özata *et al*. 2011). Authentication of the cell line was verified by short tandem repeat profiling, as described in our recent study (Özata *et al*. 2011).

### DNA and RNA extraction

Genomic DNA was extracted using DNeasy Blood and Tissue Kit (Qiagen) and total RNA was isolated using mirVana miRNA Isolation Kit (Applied Biosystems/Ambion, Austin, TX, USA). Quantification of isolated DNA or RNA was performed by NanoDrop ND-1000 spectrophotometer (NanoDrop Technologies, Wilmington, DE, USA).

### RT quantitative real-time PCR (RT-qPCR) analysis

cDNA was synthesized from 100 ng of total RNA using High Capacity cDNA RT Kit (Applied Biosystems), and the mRNA expression was quantified for *DICER* (*DICER1*) (ID_00998578_m1), *DROSHA* (ID_01095029_m1), *TARBP2* (ID_00998379_m1), *DGCR8* (ID_00987089_m1), *PRKRA* (ID_00269379_m1), *IGF2* (ID_00277496_s1), and *H19* (ID_00399293_g1) using a 7900HT Real-Time PCR System (Applied Biosystems). Expression of *18S* rRNA (ID_99999901_s1) was evaluated in parallel for normalization purpose. *18S* was chosen as a reference gene for its stable expression in all samples (Supplementary Figure S1, see section on supplementary data given at the end of this article). Expression levels of *miR-497* (ID_001043) and *miR-195* (ID_000494) were quantified in the NCI-H295R cells after transfection experiments to evaluate transfection efficiency. The expression of these two miRNAs has previously been evaluated in a subset of the adrenocortical tumors (Özata *et al*. 2011). Here, we analyzed the levels of *miR-195* and *miR-497* in additional five ACC cases included in this study. Normalization for miRNA expression was done using *RNU6B* (*RNU6-6P*) (ID_001093). All reactions were performed in triplicate, and relative expression levels were reported as 2^−^^Δ^^*C*T^.

### Western blot analysis

Whole cell lysates were prepared as described previously (Özata *et al*. 2011) for detection of miRNA machinery proteins using primary antibody anti-TARBP2 (sc-100909; Santa Cruz Biotechnology, Inc.) at 1:1000 dilution, anti-DICER (ab14601; Abcam, Cambridge, UK) at 1:200 dilution, or anti-DROSHA (ab12286; Abcam) at 1:400 dilution. Anti-GAPDH antibody (sc-47724; Santa Cruz Biotechnology, Inc.) diluted at 1:5000 was used for normalization. Protein levels were quantified on X-ray films from immunoblots using ImageJ software (http://rsb.info.nih.gov/ij/).

### Immunohistochemistry

Immunohistochemistry of TARBP2 was performed in 34 adrenocortical tumors (17 adenomas and 17 ACCs) and five normal adrenal tissues using anti-TARBP2 (ab72547, Abcam). The details are available in the Supplementary Materials and methods, see section on supplementary data given at the end of this article. The TARBP2 immunostaining and subcellular localization were evaluated by a pathologist (A H). The cytoplasmic immunoreactivity was expressed in most tumor cells with the most obvious difference in the intensity of the immunoreactivity. Therefore, the clinical specimens were scored as having strong, moderate, or weak/negative TARBP2 immunoreactivity. The nuclear immunoreactivity, on the other hand, did not appear in all nuclei and the cases were classified as positive, mixed, or negative. In the adenomas, two different cell types were identified (oxyphilic and lipid-rich cells) and evaluated for both cytoplasmic and nuclear TARBP2 staining.

### Validation of potential molecular biomarkers in independent cohorts

Expression data from two previously published microarray data sets were downloaded from ArrayExpress database. Giordano *et al*.'s study included a total of 55 adrenocortical tumors (22 adenomas and 33 carcinomas) and ten adrenal cortices (http://www.ebi.ac.uk/arrayexpress/experiments/E-GEOD-10927), while 92 adrenocortical tumors (58 adenomas and 34 carcinomas) were analyzed in de Reynies *et al*.'s study (http://www.ebi.ac.uk/arrayexpress/experiments/E-TABM-311). Expression values for *TARBP2* (HG-U133_Plus_2.0 probe set: 203677_s_at), *DICER* (213229_at, 206061_s_at), *DROSHA* (2218269_at), *IGF2* (202409_at, 202410_x_at, 210881_s_at), and *H19* (224646_x_at, 224997_x_at) were extracted from the microarray data. Geometrical mean was used to obtain a single expression value for genes that were represented by more than one probe set on the Affymetrix chip. Comparison of mRNA expression among sample groups for each gene and evaluation of their predictive value in ACC classification was performed as described in the ‘Statistical analyses’ section.

### Transfection experiments in NCI-H295R cells

NCI-H295R cells were transfected using Amaxa Nucleofector technology (Lonza, Basel, Switzerland) with pre-miR-195, pre-miR-497 (PM10827 and PM10490 respectively; Applied Biosystems/Ambion), or siTARBP2 (sc-106846; Santa Cruz Biotechnology, Inc.), as described previously (Özata *et al*. 2011). Pre-miR Negative control#1 (4464058; Applied Biosystems/Ambion) or siCTR (sc-36869; Santa Cruz Biotechnology, Inc.) were used as negative controls. All transfection experiments were repeated at least three times.

### WST-1 colorimetric assay

Cell viability was evaluated using WST-1 colorimetric assay (Roche Applied Science), as described previously (Özata *et al*. 2011). All experiments were conducted in eight wells for each condition and replicated at least three times independently. Cell viability was calculated by comparing the absorbance values of the samples after background subtraction and normalized to the siCTR-treated cells.

### Apoptosis caspase-3 colorimetric assay

After 72 h of transfection, the effect on apoptosis was evaluated using caspase-3 colorimetric assay (Genscript, Piscataway, NJ, USA), as described previously (Özata *et al*. 2011). Relative caspase-3 activity was calculated by comparing the absorbance values of the siTARBP2-treated cells with the respective siCTR-treated cells. All experiments were replicated three times.

### Mutation analysis

All coding exons and flanking exon–intron junctions of the *TARBP2* gene (NM_134323) were sequenced in 23 ACC specimens. The PCR products were purified using ExoSAP-IT (USB Corporation/Affymetrix, Cleveland, OH, USA) and sequenced at the KIGene facility. Primer sequences and PCR conditions used for the analysis are detailed in Supplementary Table S1, see section on supplementary data given at the end of this article.

### TaqMan copy number assay

TaqMan copy number assay (Applied Biosystems) was used to evaluate the changes of *TARBP2* copy number in adrenal cortices and adrenocortical tumors. The target gene *TARBP2* (ID_02091089_cn) and the reference gene *RNaseP* (ID_4403326) were analyzed in parallel. The calculated relative copy numbers and the predicted copy numbers were estimated using CopyCaller software (Applied Biosystems). DNA from normal adrenal cortex was used as calibrator for the analysis. All reactions were performed in triplicate.

### Argonaute 2 co-immunoprecipitation and analysis of argonaute 2-associated mRNAs

Cells (2×10^6^ cells/dish) were transfected with pre-miR Negative control#1, pre-miR-195 or pre-miR-497, and seeded in six tissue culture plates (10 cm). After 72 h of transfection, the cells were used for co-immunoprecipitation (co-IP) experiments using protein G Sepharose 4 Fast Flow beads (17-0618-01; GE Healthcare, Uppsala, Sweden) coated with mouse anti-human argonaute 2 (Ago2) antibody (ab57113; Abcam), as described previously (Xie *et al*. 2012). Ago2-bound RNA was extracted with TRIzol reagent (Invitrogen). *DICER* and *TARBP2* mRNA expression levels were measured by RT-qPCR and normalized to *miR-483-3p* for input and IP samples. This miRNA was chosen as an internal control due to its high abundance in the NCI-H295R cells. Enrichment of *DICER* and *TARBP2* mRNAs bound to Ago2 was calculated from the relative amount of mRNA detected in IP samples divided by the relative amount of mRNA in the corresponding input samples.

### Statistical analyses

Statistica 8.0 (StatSoft, Inc., Tulsa, OK, USA) or MS Office Excel was used for statistical calculations. One-way ANOVA and unpaired Student's *t*-test were used to compare mRNA and protein expression levels among or between sample groups, and paired Student's *t*-test was performed to analyze transfection and Ago2-IP experiments. The patient population was divided into two groups based on high or low expression of *DICER*, *DROSHA*, *TARBP2*, *IGF2*, or *H19* according to the median expression levels among the adrenocortical tumors. Correlations between *TARBP2*, *DICER*, and *DROSHA* mRNA and protein expression levels were assessed by Pearson's correlation analyses and *P* values were estimated by permuting the samples 1000 times. The association between mRNA gene expression levels and clinical–histopathological parameters was analyzed using *χ*^2^ test. Kaplan–Meier analysis was carried out to obtain survival curves. The survival curves of different patient groups were compared using log-rank test. Overall survival was the period from the time of diagnosis until the end of follow-up or death of the patient. For overall survival analysis, survival was censored if patients were still alive at the end of the follow-up. Disease-free survival was the time between the initial diagnosis and the end of follow-up or documented recurrence/death. For disease-free survival analysis, the data were censored if patients did not show any recurrence or died for other causes not related to disease. In the survival analyses, the recurrent ACC (Ca30) was not included. All the analyses were two tailed and *P* values <0.05 were considered significant.

## Results

### Deregulation of miRNA-processing factors in adrenocortical tumors and clinical associations

**Figure 1:**
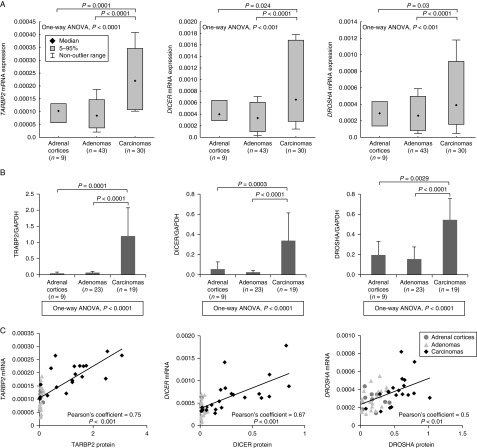
Relative expression levels of miRNA-processing machinery components in adrenocortical tumors and adrenal cortices. (A) Box plots show relative mRNA expression levels for *TARBP2*, *DICER*, and *DROSHA* in nine normal adrenal cortices, 43 adenomas, and 30 carcinomas measured by RT-qPCR. *18S* was used as a reference gene. The expression levels of mRNA between groups were compared using one-way ANOVA models and *P*<0.05 was considered significant. (B) Comparison of TARBP2, DICER, and DROSHA protein expression levels among different groups. GAPDH was used for normalization. *P* values were calculated using one-way ANOVA models and *P*<0.05 was considered significant. (C) Scatter plots showing correlation between mRNA and protein levels for TARBP2, DICER, and DROSHA as assessed by Pearson's correlation analysis.

We analyzed the mRNA expression levels of five miRNA-processing genes (*DROSHA*, *DGCR8*, *DICER*, *TARBP2*, and *PRKRA*) in 73 adrenocortical tumors and nine adrenal cortices using RT-qPCR. In comparison to adrenal cortices, 29 of 30 ACCs (96%) showed a significantly increased expression of *TARBP2* (*P*=0.0001). Twenty-six carcinomas (87%) also exhibited significant over-expression of *DICER* (*P*=0.024) and 24 (80%) of the carcinomas presented a significantly increased *DROSHA* expression (*P*=0.03) (Fig. 1A). By contrast, abnormal mRNA expression of *TARBP2*, *DICER*, and *DROSHA* was less frequent in adenomas (*TARBP2*, 32% (14/43); *DICER*, 37% (16/43); and *DROSHA*, 40% (17/43)) (Fig. 1A). Significant over-expression of *TARBP2* (1.3- to 4-fold, *P*<0.0001), *DICER* (0.4- to 5-fold, *P*<0.0001), and *DROSHA* (0.2- to 4.5-fold, *P*<0.0001) was also observed in carcinomas when compared with adenomas (Fig. 1A). However, we did not observe any significant differences for *DGCR8* or *PRKRA* between the three sample groups (Supplementary Figure S2, see section on supplementary data given at the end of this article).

The adenoma samples included in the study consisted of 13 Cushing, 16 aldosteronoma, and 14 non-hyperfunctional tumor samples. Comparing the mRNA expression levels of the five miRNA machinery genes analyzed, we did not detect any significant differences between the three adenoma subgroups (Supplementary Figure S3). Moreover, for carcinoma cases, we did not observe any significant association between *DROSHA*, *DGCR8*, *DICER*, *TARBP2*, or *PRKRA* mRNA levels and clinical–histopathological parameters such as gender, age, tumor size, or presence of metastasis (data not shown).

To establish whether the increased mRNA expressions were reflected on the protein level, TARBP2, DICER, and DROSHA were examined by western blot analysis in a subset of nine adrenal cortices, 23 adenomas, and 19 carcinomas. Concordantly with gene expression results, carcinoma cases showed a significant increased expression of the three proteins analyzed when compared with adenomas and adrenal cortices (*P*<0.01 for all comparisons) (Fig. 1B and Supplementary Figure S4, S5, and S6). In addition, Pearson's correlation analysis showed a significant correlation between western blot and RT-qPCR results for TARBP2 (*Cor*=0.75, *P*<0.001), DICER (*Cor*=0.67, *P*<0.001), and DROSHA (*Cor*=0.5, *P*<0.01) (Fig. 1C).

Sixteen of the ACC cases included in the study were diagnosed with distant metastases (seven at time of diagnosis and nine during follow-up). Among the ACCs, univariate analysis identified presence of distant metastasis as a predictive factor of shorter overall survival (log-rank, *P*=0.014) and disease-free survival (log-rank, *P*=0.001) (Supplementary Figure S7). However, we did not find any significant association between size, gender, age, or expression levels of miRNA-processing factors with survival among the ACC patients (data not shown).

### Immunohistochemical analysis of TARBP2 expression

**Figure 2:**
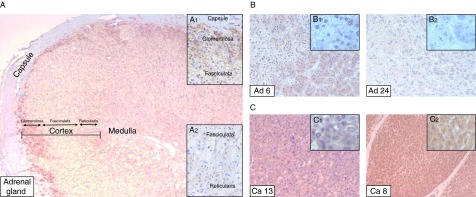
Immunohistochemical detection of TARBP2 expression in normal adrenal glands (A, ×40), adrenocortical adenomas (B, ×160), and adrenocortical carcinomas (C, ×160). (A) In the normal adrenal gland, TARBP2 staining is mainly present in the zona glomerulosa and, at lower intensity, in the zona fasciculata and reticularis (inserts A_1_ and A_2_, ×160). (B) The adenomas show a moderate (insert B_1_, ×400) or weak/negative (insert B_2_, ×400) TARBP2 expression in the cytoplasm of oxyphilic cells while lipid-rich cells only demonstrate weak/negative cytoplasmic TARBP2 staining. Most of the adenomas showed positive or mixed nuclear TARBP2 immunoreactivity among the oxyphilic cells and negative TARBP2 expression in the nuclei of lipid-rich cells. (C) The majority of carcinomas show a strong TARBP2 expression in the cytoplasm and mainly positive (insert C_1_, ×400) or mixed (insert C_2_, ×400) nuclear staining.

Based on the RT-qPCR and western blot analyses, TARBP2 showed the strongest upregulation in ACC. To further confirm this over-expression, we evaluated the protein level of TARBP2 in a subset of adrenocortical tumors (17 adenomas and 17 carcinomas) and five normal adrenal glands using immunohistochemistry. The immunostaining results are summarized in Supplementary Table S2 and exemplified in Fig. 2.

RNA interference (RNAi) activity mainly occurs in the cytoplasm, and the essential RNAi-processing factors, such as DICER, TARBP2, and argonautes, are usually localized in the cytoplasm (Chendrimada *et al*. 2005). Interestingly, Kim *et al*. (2006) reported that TARBP2 is required for siRNA-mediated transcriptional silencing in human cells, suggesting its role in the nuclear compartment. We therefore sought to determine subcellular localization and expression of TARBP2 in adrenocortical tumors. Our results showed that TARBP2 was mainly expressed in the cytoplasm, although a weaker nuclear staining was also observed. In the normal adrenal tissues, positive cytoplasmic staining for TARBP2 was present in cells of the zona glomerulosa while cells of zona fasciculata and reticularis showed weak/negative cytoplasmic staining (Fig. 2A). In the adenomas, TARBP2 staining was mainly confined to oxyphilic cells while the preponderance of lipid-rich cells was negative for TARBP2 immunoreactivity. Twelve of the 17 adenomas (70%) showed a moderate cytoplasmic staining, four cases (24%) demonstrated weak or negative TARBP2 expression while only one case presented strong TARBP2 immunoreactivity (Fig. 2B and Supplementary Table S2). Concordantly with the results obtained by RT-qPCR and western blot, most of the ACCs (14/17, 82%) showed strong cytoplasmic TARBP2 staining in nearly all tumor cells, while moderate TARBP2 expression was detected in only three ACCs (18%) (Fig. 2C and Supplementary Table S2).

At nuclear level, among the five normal adrenal cortices, only a single case showed a mixed nuclear staining in the cells of the zona glomerulosa, while all the other samples exhibited negative TARBP2 nuclear immunoreactivity. Among the adenomas, most of the cases (12/17) presented mixed or positive nuclear TARBP2 expression in oxyphilic cells while the remaining five cases showed negative staining in the nuclei of the same cell type. On the other hand, lipid-rich cells had mixed or positive nuclear staining in five adenomas and negative in 12 adenomas. The majority of ACC tumor cells were positive for TARBP2 nuclear expression (10/17), while five showed mixed nuclear staining and two cases were negative (Fig. 2 and Supplementary Table S2). However, positive, mixed, or negative TARBP2 nuclear expression did not show any significant correlation with histopathological parameters and survival among the ACC cases (data not shown).

### *TARBP2* as a novel molecular predictor of carcinoma

Given the observed over-expression of *TARBP2*, *DICER*, and *DROSHA* in ACCs, we sought to investigate whether the ACCs could be accurately classified based on the expression levels of these three genes. Adrenocortical tumor patients were divided into high or low mRNA levels of *TARBP2*, *DICER*, and *DROSHA* according to their median expression levels. Among the three genes analyzed, *TARBP2* showed the highest sensitivity and specificity to discriminate between ACCs and adenomas. Twenty-seven patients with ACCs were correctly predicted based on the expression of *TARBP2* mRNA (27/29 patients, 93% sensitivity); however, 10/43 adenoma cases were misclassified, leading to a specificity of 76%. Classification of ACCs based on *DICER* or *DROSHA* expression levels resulted in a much lower sensitivity and specificity (76 and 67% respectively).

Previous studies have clearly shown over-expression of IGF2 (Gicquel *et al*. 2001, Giordano *et al*. 2009, Laurell *et al*. 2009) or decreased expression of *H19* (Gao *et al*. 2002, Giordano *et al*. 2009) in ACCs and demonstrated the potential of IGF2 as a biomarker for ACC diagnosis (Gicquel *et al*. 2001). We therefore compared the predictive values of *IGF2*, *H19*, *TARBP2*, *DICER*, and *DROSHA* for detection of ACC. As shown in Supplementary Table S3, see section on supplementary data given at the end of this article, the classification based on *H19* and *TARBP2* expression had similar levels of sensitivity (SEN=93% for both *H19* and *TARBP2*) and specificity (SPE=79% for both *H19* and *TARBP2*), while *IGF2* showed a slightly lower sensitivity (89%) and specificity (77%) when compared with *TARBP2*. The combination of *TARBP2*, *IGF2*, and *H19* expressions had an additive effect in the predictive value for ACC (SEN=97% and SPE=81%). On the other hand, both *DICER* and *DROSHA* had lower prediction power than the three genes described earlier. The sensitivity, specificity, positive predictive value, negative predictive value, and overall accuracy for the five genes analyzed are summarized in Supplementary Table S3.

**Figure 3:**
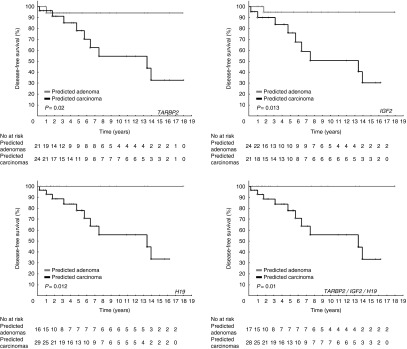
Prediction of carcinoma based on the mRNA expression levels of *TARBP2*, *IGF2*, and/or *H19*. The Kaplan–Meier curves separate the patients according to the expression levels of the molecular predictors *TRBP2*, *IGF2*, and/or *H19*. The 45 clinical cases were divided in two groups with high or low expression levels of *TARBP2*, *IGF2*, or *H19* according to their median expression levels measured by RT-qPCR. A case was predicted as carcinoma if the expression level was >0.00012 for *TARBP2*, >0.00025 for *IGF2*, <0.0019 for *H19*, or >−0.0013 for combination of the three genes. The curves of each group were compared using log-rank test and *P*<0.05 was considered significant.

To further investigate the diagnostic power of *TARBP2*, *DICER*, and *DROSHA* in adrenocortical tumors and overcome limitations of pathological assessment, we applied disease-free survival as the end point for diagnosing ACC, which is similar to the approach applied by de Reynies *et al*. (2009). Among the 72 tumors analyzed, *TARBP2* mRNA expression was a good predictor of ACC (log-rank, *P*<0.001) and allowed an accurate classification of all seven cases with metastasis at the time of diagnosis. *DROSHA* could also significantly predict ACC cases (log-rank, *P*=0.038), although two carcinoma cases presenting metastasis at diagnosis were misclassified. However, *DICER* did not show any statistical significance (Supplementary Figure S8). We also tested the predictive potential for *TARBP2* and *DROSHA* to detect ACCs among the 45 potentially malignant adrenocortical tumors after exclusion of tumors with sizes ≤3 cm and the seven patients presenting metastases at diagnosis. As shown by Kaplan–Meier analysis, *TARBP2* was still found highly predictive of ACC among those without metastases at diagnosis (log-rank, *P*=0.02; Fig. 3) while *DROSHA* did not show any statistical significance (data not shown). The classification obtained according to *TARBP2* expression was at least as accurate as the prediction based on *IGF2* and *H19* expressions or the combination of the three genes (Fig. 3).

### Validation in independent cohorts of adrenocortical tumors

To strengthen our findings, we studied independent cohorts of 147 adrenocortical tumors and ten normal adrenal cortices from two previously published microarray data sets (Giordano *et al*. 2009, de Reynies *et al*. 2009). Concordantly with our results, *TARBP2*, *DICER*, and *DROSHA* were significantly over-expressed in carcinomas when compared with adenomas or normal adrenal cortices in both studies (Supplementary Figure S9, see section on supplementary data given at the end of this article). Moreover, in both cohorts, we confirmed the higher predictive values for *TARBP2* when compared with *DICER* or *DROSHA* in the classification of ACC among adrenocortical tumors (Giordano *et al*.'s cohort: *TARBP2*, SEN=84%, SPE=100%; de Raynies *et al*.'s cohort: *TARBP2*, SEN=80%, SPE=82%). As observed in our cohort, the combination of *TARBP2*, *IGF2*, and *H19* could improve the overall prediction for ACC classification in both data sets (Supplementary Tables S4 and S5, see section on supplementary data given at the end of this article).

### TARBP2 affects cell viability and apoptosis in NCI-H295R cells

**Figure 4:**
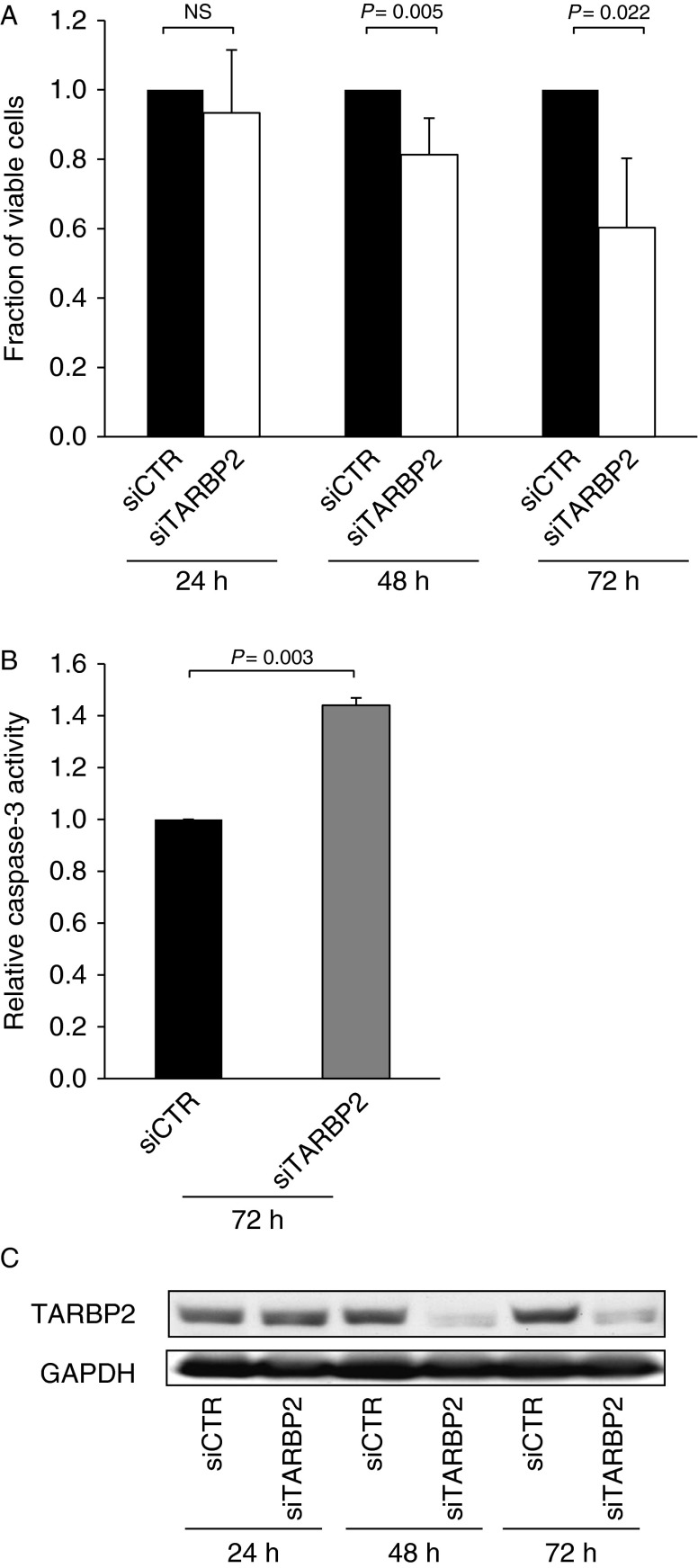
Silencing of *TARBP2* in NCI-H295R cell line affects cell viability and apoptosis. (A) Histogram showing fractions of viable cells measured by WST-1 colorimetric assay in NCI-H295R cells transfected with siTARBP2 or siCTR at different time points (24, 48, and 72 h). (B) Histogram illustrating the increase of cell death evaluated by caspase-3/CPP32 colorimetric assay in NCI-H295R cells transfected with siTARBP2 when compared with siCTR-transfected cells. Error bars represent s.d. of the mean of three independent experiments. Paired *t*-test was used to determine the differences between groups and *P*<0.05 were considered significant. (C) A representative western blot showing TARBP2 levels at 24, 48, and 72 h after transfection with siTARBP2 or siCTR. Incubation with a GAPDH antibody was used as a loading control.

The over-expression of TARBP2 observed in ACC tumors prompted us to investigate the functional consequences of TARBP2 alteration in human NCI-H295R ACC cells. Upon inhibition of TARBP2 expression, ACC cells showed a significant reduction of cell viability over time (Fig. 4A) and a markedly increase (∼30%) of cell death (Fig. 4B) in comparison with negative control cells.

### *TARBP2* sequencing and copy number variations

We investigated possible mechanisms that could induce over-expression of TARBP2 in ACCs. First, genomic DNA from 23 ACCs was screened for *TARBP2* gene mutations by sequencing. However, no mutations were identified in any of the coding exons analyzed (data not shown).

As the *TARBP2* gene is located in the chromosomal region 12q13.13 that is frequently gained/amplified in ACC (Stephan *et al*. 2008), we investigated *TARBP2* gene copy number alterations in a subset of 28 carcinomas, 18 adenomas, and 3 normal adrenal cortices using TaqMan copy number assay. We observed copy number gain in 57% of the carcinoma cases (16/28), but no changes among the adenomas and normal tissue samples (Supplementary Figure S10, see section on supplementary data given at the end of this article). However, the genomic copy number alteration did not always correspond with the *TARBP2* mRNA levels. For examples, four tumors (Ca3, Ca20, Ca22, and Ca25), showing *TARBP2* copy number gain, were among the cases with relatively low *TARBP2* expression (Supplementary Figure S10). This discrepancy implies that additional mechanism(s), e.g. posttranscriptional regulation by miRNAs, may involve in TARBP2 regulation.

### *miR-195* and *miR-497* regulate *TARBP2* and *DICER* expression in adrenocortical tumors

To explore a possible involvement of miRNAs in TARBP2 regulation, we first searched for the published under-expressed miRNAs in ACCs, thus showing an inverse expression pattern compared with *TARBP2*. We found 12 downregulated miRNAs that were reported in more than one study (Soon *et al*. 2009, Tombol *et al*. 2009, Özata *et al*. 2011, Patterson *et al*. 2011, Schmitz *et al*. 2011; Supplementary Table S6, see section on supplementary data given at the end of this article).

**Figure 5:**
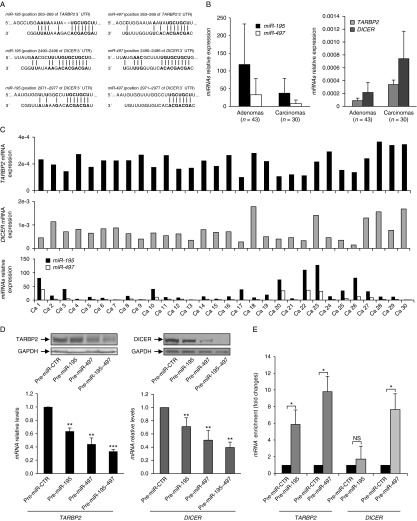
*miR-195* and *miR-497* target *TARBP2* and *DICER*. (A) Schematic presentation of *miR-195* and *miR-497* predicted target sites identified in the *TARBP2* and *DICER* 3′-UTRs by TargetScan 5.2. (B) *miR-195*/*miR-497* and *TARBP2*/*DICER* mRNA relative expression levels in adrenocortical carcinomas (*n*=30) in comparison to adenomas (*n*=43) assessed by RT-qPCR. The two miRNAs show an inverse pattern of expression compared with *TARBP2* and *DICER*. (C) Comparison between *miR-195*/*miR-497* and *TARBP2*/*DICER* mRNA expression levels in adrenocortical carcinoma cases measured by RT-qPCR. (D) Transfection of pre-miR-195 and/or pre-miR-497 in NCI-H295R cell line represses mRNA and protein expression of TARBP2 and DICER when compared with pre-miR-CTR. (E) NCI-H295R cells were transfected with pre-miR-CTR, pre-miR-195, or pre-miR-497. The levels of Ago2-associated *TARBP2* and *DICER* mRNAs were measured by RT-qPCR relative to *miR-483-3p*. The mRNA enrichment of *TARBP2* or *DICER* in each anti-Ago2 IP sample was normalized to the corresponding input sample. The results are shown as fold change in comparison to the negative control and the error bars represent s.d. of the mean of three independent experiments. Differences in expression levels between groups were evaluated using paired *t*-test and *P*<0.05 was considered significant. *P<0.05; **P<0.01; ***P<0.001; NS, not significant.

Next, we performed bioinformatic analysis using TargetScan (http://www.targetscan.org/) to identify whether any of the downregulated miRNAs have potential binding sites in the 3′-UTR of *TARBP2*. The analysis identified *let-7* family members, *miR-195* and *miR-497*, as possible regulators of *TARBP2*. In addition, potential target sites for these miRNAs were also present in the 3′-UTR of *DICER* (Fig. 5A). As *let-7* family members were not significantly deregulated in ACCs compared with adenomas in our cohort (Özata *et al*. 2011), we focused on *miR-195* and *miR-497*. These miRNAs belong to the same miRNA cluster and show an inverse expression pattern in comparison with *TARBP2* and *DICER* expression levels (Fig. 5B). Noteworthy, the four carcinomas with *TARBP2* copy number gain and low *TARBP2* expression exhibited high expression of *miR-195* and *miR-497*. The same cases also presented low levels of *DICER* (Fig. 5C). These observations prompted us to investigate the role of *miR-195* and *miR-497* as potential regulators of *TARBP2* and/or *DICER*.

To confirm whether *TARBP2* and *DICER* were biological targets of *miR-195* and *miR-497* in adrenocortical tumors, we transfected NCI-H295R cells with miRNA mimics (pre-miR-195 and/or pre-miR-497). We observed a significant reduction of TARBP2 and DICER mRNA and protein expression levels in the cells over-expressing *miR-195* or *miR-497* compared with the cells transfected with pre-miR-negative control. *miR-497* had a stronger repressive effect on both TARBP2 and DICER, and the combined over-expression of the two miRNAs showed a synergic effect on posttranscriptional regulation (Fig. 5D).

Next, we adopted the Ago2-immunoprecipitation approach to determine whether *TARBP2* and *DICER* are direct targets of *miR-195* and/or *miR-497*. Previous studies have shown that Ago complexes can stably associate with miRNA targets (Beitzinger *et al*. 2007, Karginov *et al*. 2007). Thus, we immunoprecipitated Ago2 complexes in NCI-H295R cells after induced over-expression of *miR-195* or *miR-497* and measured the abundance of the co-immunoprecipitated *TARBP2* and *DICER* mRNAs by RT-qPCR. We observed a significant enrichment of endogenous mRNAs for both genes in cells over-expressing *miR-497* (*P*<0.05). *TARBP2* mRNA was also strongly enriched in the immunoprecipitates of *miR-195* over-expressing cells (*P*<0.05) while for *DICER* mRNA the enrichment did not show any statistical significance (Fig. 5E).

## Discussion

We report dysregulation of miRNA machinery components in adrenocortical tumors and the potential role of *TARBP2* as molecular biomarker for ACC classification. In addition, we show the mechanisms of TARBP2 regulation and the functional consequences of TARBP2 deregulation in ACC cells.

### Over-expression of TARBP2, DICER, and DROSHA in ACCs

We show that TARBP2, DICER, and DROSHA are significantly over-expressed in ACC when compared with adenomas and adrenal cortices. The expression pattern was validated in publicly available data sets. Disruption of miRNA machinery has been previously associated with tumorigenesis in several cancer types. In line with our findings, over-expression of *TARBP2* and *DICER* was observed in prostate cancer (Fu *et al*. 2010). DICER over-expression was also reported in cutaneous melanomas (Ma *et al*. 2011, Sand *et al*. 2011) and was associated with aggressiveness in lung adenocarcinoma (Chiosea *et al*. 2007) and poor survival in colorectal cancer (Faber *et al*. 2011). Higher expression of DROSHA was found in cervical squamous cell carcinomas (Muralidhar *et al*. 2007) and epithelial skin cancers (Sand *et al*. 2010). Its over-expression was associated with poor prognosis in esophageal cancer (Sugito *et al*. 2006). On the contrary, reduced expression of miRNA-processing factors has also been observed in several tumor types. Decreased expression of TARBP2 was reported in colorectal and gastric cancers presenting microsatellite instability (Melo *et al*. 2009). Low expression of DROSHA and DICER was associated with decreased survival in ovarian cancer (Merritt *et al*. 2008). Decreased mRNA level of *DICER* was observed in basal cell carcinomas (Sand *et al*. 2010) and was also associated with poor prognosis in non-small-cell lung cancer (Karube *et al*. 2005). Together, variation of TARBP2, DICER, and DROSHA expression levels among different tumor types suggests that deregulation of miRNA-processing factors can be dependent on cellular context and imply their possible dual role as tumor suppressors or oncogenes in human cancers. Despite that we have not investigated whether deregulation of miRNA machinery genes affects the miRNA-processing efficiency in ACC cells, these effects have been demonstrated for TARBP2 and DICER in colorectal and breast cancer cells respectively (Melo *et al*. 2009, 2011, Martello *et al*. 2010).

Interestingly, we observed that TARBP2 was localized both in the cytoplasm and nucleus of tumor cells, whereas its expression was mainly found in the cytoplasm of non-tumor cells. In line with our findings, RNAi pathway components (including DICER, TARBP2, AGO1, and AGO2) have been observed in the nuclear compartment of human cells (Haussecker & Proudfoot 2005, Kim *et al*. 2006, Ahlenstiel *et al*. 2012). These observations suggest that TARBP2 may involve in nuclear RNAi on transcriptional silencing or other unknown function(s). Further analyses are warranted to investigate the potential nuclear role of TARBP2 and its implication in the pathogenesis of adrenocortical tumors.

### *TARBP2* as molecular predictor of carcinoma and functional role in ACC cells

Discrimination between localized ACCs and adenomas is challenging. To overcome these clinical limitations, the identification of novel molecular biomarkers is certainly needed. In our study, we show that *TARBP2* is a strong predictor of carcinoma and can reliably classify ACC in a cohort of non-metastatic adrenocortical tumors.

Consistent with the over-expression observed in ACC, we also demonstrated that inhibition of TARBP2 expression could affect cell growth and apoptosis in the NCI-H295R cells. In line with these results, previous studies demonstrated that TARBP2 promotes cell growth and transformation *in vitro* and can induce tumor formation in mice (Benkirane *et al*. 1997, Lee *et al*. 2004). These findings suggest a possible oncogenic function of TARBP2 in ACC carcinogenesis and imply the potential use of TARBP2 as a novel therapeutic target in ACC.

### Regulatory mechanisms of TARBP2 over-expression in ACC

Despite several studies showing alteration of miRNA machinery factors in different tumor types, the molecular mechanisms that regulate their expression are not fully understood. Given the over-expression and the potential oncogenic role of TARBP2 in ACC, we sought to investigate possible molecular mechanisms involved in the regulation of TARBP2 expression. *TARBP2* gene mutations causing a loss of TARBP protein expression have been previously shown in colorectal and gastric cancers (Melo *et al*. 2009). However, in our study, the mutational analysis revealed the presence of only wild-type sequences for the *TARBP2* gene in all the ACC cases analyzed, implying that genomic DNA mutations are probably not involved in the deregulation of TARBP2 in ACC. On the other hand, we found *TARBP2* gene copy number gain in 57% (16/28) of the carcinomas, suggesting that copy number gain of *TARBP2* gene may be, at least partially, responsible for its over-expression in ACC.

In addition, we propose a miRNA-mediated regulation of *TARBP2* expression in ACC. While there is no evidence in the literature of miRNAs affecting TARBP2 expression, *let-7* and *miR-103*/-*107* family are known to regulate expression of DICER (Forman *et al*. 2008, Martello *et al*. 2010). Here, we demonstrate that mature *miR-195* and *miR-497* can directly regulate *TARBP2* and *DICER* expression in ACC. Downregulation of *miR-195* and/or *miR-497* has been observed in several tumor types, including ACC (Soon *et al*. 2009, Özata *et al*. 2011, Patterson *et al*. 2011, Schmitz *et al*. 2011), liver (Xu *et al*. 2009), bladder (Han *et al*. 2011), breast (Li *et al*. 2011), and peritoneal carcinoma (Flavin *et al*. 2009). Reduced expression of *miR-195* is also correlated with lymph node metastasis and poor prognosis in colorectal cancer (Wang *et al*. 2011). In line with the expression pattern, we previously showed that over-expression of *miR-195* and *miR-497* can inhibit cell growth with concomitant increase of apoptosis in NCI-H295R ACC cells (Özata *et al*. 2011). Together, it is intriguing to speculate that the phenotypic effect observed in ACC cell line upon alteration of *miR-195* and *miR-497* expression may be mediated through TARBP2 and DICER downregulation.

In summary, we report frequent over-expression of TARBP2 in ACC and we found that *TARBP2* mRNA expression level is a useful predictor of ACC and able to discriminate adenomas from carcinomas. In addition, we revealed the potential oncogenic role of TARBP2 and mechanisms of its over-expression in ACC.

## Supplementary data

This is linked to the online version of the paper at http://dx.doi.org/10.1530/ERC-13-0098.

## Author contribution statement

S Caramuta and W-O Lui conceived and designed the experiments; S Caramuta and L Lee developed the methodology; S Caramuta, L Lee, D M Özata, P Akçakaya, and H Xie performed the experiments; S Caramuta, L Lee, D M Özata, P Akçakaya, H Xie, A Höög, and W-O Lui analyzed and interpreted the data; S Caramuta, L Lee, D M Özata, P Akçakaya, H Xie, A Höög, J Zedenius, M Bäckdahl, C Larsson, and W-O Lui contributed to write and/or revise the manuscript; S Caramuta, L Lee, J Zedenius, M Bäckdahl, C Larsson, and W-O Lui contributed to administrative, technical, or material support; S Caramuta and W-O Lui supervised the study.

## Figures and Tables

**Table 1:** Clinical features for the adrenocortical tumor cases studied

**Clinical features**	**Adenomas**	**Carcinomas**
No. of cases	43	29
Gender		
Male	12	13
Female	31	16
Age (years)		
Median	55	61
Min–max	16–81	28–84
Subtypes		
Cushing	13	NA
Aldosteronoma	16	NA
Non-hyperfunctioning	14	NA
Tumor size (cm)^a^		
Median	3.5	12
Min–max	0.9–6.5	7–21
Metastasis		
Yes	0	16
No	43	13
Follow-up (months)		
Median	39	42
Min–max	12–258	2–195
Follow-up (outcome)		
Alive	38	13
Dead of other causes^b^	5	4
DOD	0	12

NA, not available; DOD, dead of disease.

a No information was available for one of the carcinomas.

b Patients who died for causes not related to the disease.
